# A Case of Syncronised Hereditery Nonpoliposis Colorectal Tumor With Different Hystopathological Type and K-Ras Gene Mutation: Case Report

**DOI:** 10.4021/jocmr1123w

**Published:** 2013-01-11

**Authors:** Bulent Dinc, Halis Musfik Temel

**Affiliations:** a Department of General Surgery, Ataturk Station Hospital, Antalya, Turkey

**Keywords:** Colorectal neoplasms, Hereditary nonpolyposis, Synchronous neoplasms, K-ras gene

## Abstract

Hereditary nonpoliposis colorectal cancers (HNPCC) make up 2-7% of colorectal cancer (CRC) cases. CRC’s in these group of patients are usually located in proxymal colon. The incidence of syncronic metacron CRC is 18%. The incidence of having different hystopathological types for these group of tumors varies between 3-9%. APC, p53 and K-ras gene mutations may be seen in HNPCC’s. The least frequent mutation is the mutation on K-ras gene. In this report, a syncronic metacron CRC with different hystopathological type and K-ras gene mutation localised in transverse and left colon that has developed on the basis of HNPCC is discussed.

## Introduction

Colorectal cancers (CRC) with a lifetime risk of 3-5%, is most third common kinds of cancers in EU. They are the second most common cause of death due to cancers [1]. Hereditary nonpoliposis colorectal cancers (HNPCC) account for 2-7% of all CRC’s [2, 3].

HNPCC is characterised by appearing of CRC in early ages of life. Mean age for coming across to CRC is 44. Incidence of CRC appearence in whole lifetime is approximately 50-80% [4, 5]. In 60-70% of cases, CRC is located in proximal colon. The disease may be accompanied by benign or malignant extracolonic tumors (ovaries, uretera, pelvis renalis, endometrium, stomach, intestines) [6, 7]. Incidence of cancer of endometrium is 50-60% and 15% in other organs [5, 8]. Syncronised and metacron CRC incidence is 18% [9].

HNPCC tumors are poorly differanciated, medullary, mucinotic and gem ring cell tumors. Compared to sporadical tumors, poorly differanciation rate is reported to be 14% to 44% [10, 11]. According to literature, the incidence of syncronised CRC with different hystological type vary between 3-9% [12].

There are two different pathways for crc’s due to genetic mutation. These are 1) Chromosomal instability (CIN); 2) Microsatellit instability [13]. Genetic pathway in HNPCC overlaps with CIN in some way but difference arises from interference of different genes. Tumors in HNPCC pathway Express genetic mutations of APC, p53 and K-ras [14]. Mutations in K-ras are more rare than seen in CIN [15].

In this report, a metastatic syncronised CRC with different hystopathological type and K-ras mutation located in transverse and left colon is discussed.

## Case Report

A 41-year-old male resorted for an abdominal distention and intermittent abdominal pain lasting for one month. It was learned that the patient who had no complaints of defecation problems or haemotochesia, lost about 5 - 6 kg of weight after the onset of disturbances. In his family history, it was seen that his fathet died because of colon cancer at the age of 64 and that his older sister (at age 45), little sister (age 35) and two children of his mother’s brother received treatment for colon cancers. In genetic research of the patient covering Revised Amsterdam Criteria, MLH-1 germ-line mutation was determined. In physical examinatiom, a hard, fixed, palpable mass filling up the epigastric region was present. Rectal examination showed a normal sphincter tonus, a rectum full of formal faeces and no haemotochesia.

**Figure 1:**
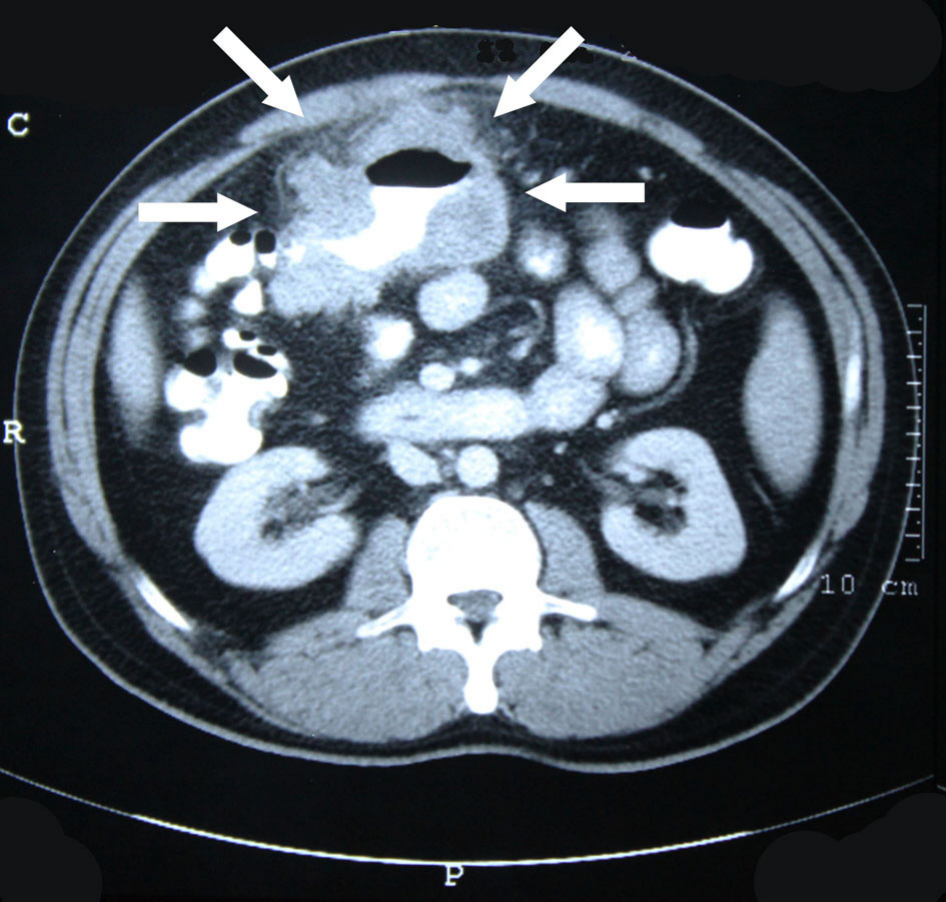
Invasive tumor at the level of transverse colon.

**Figure 2:**
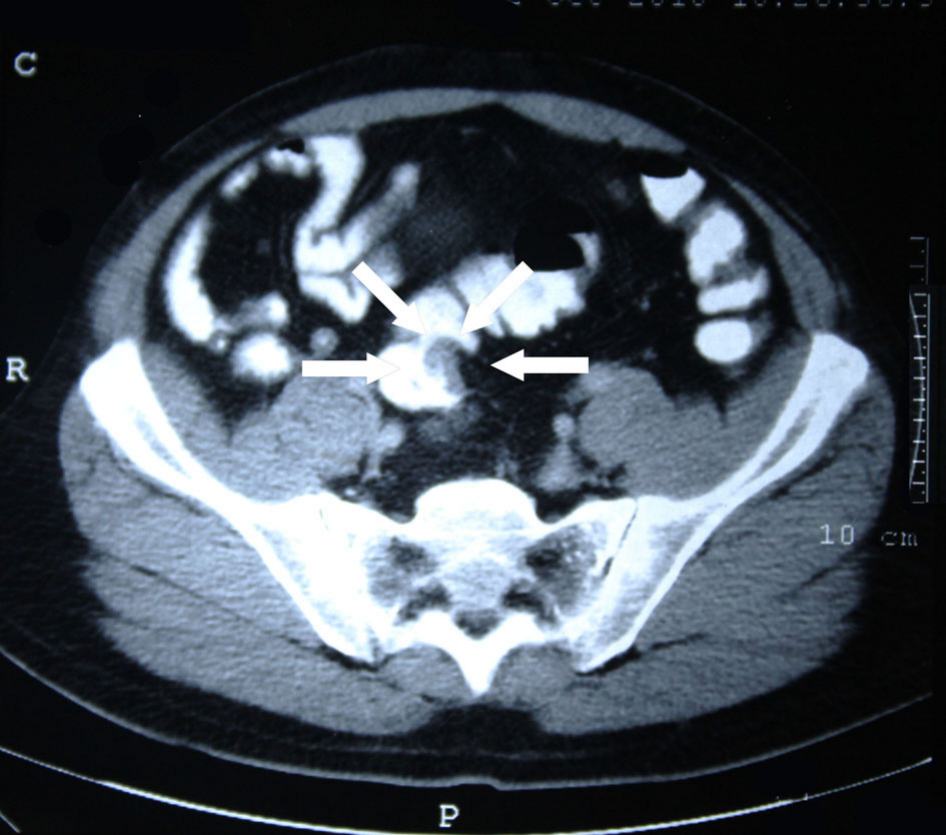
Polypoid tumor in rectosigmoid region.

In contrasted abdominal CT scan; a mass of 12 cm that shows signicant wall thickening and filling defect at transverse colon level (Fig. 1), a polypoid mass causing filling defect bulking from mucosa fitting rectosigmoidal colon in pelvis entrance (Fig. 2) were detected. No aoter intraabdominal pathological view were obtained. Colonoscopy was planned fort he patient. A polypoid mass 19 cm from anal vestibule and an invased tumor in the middle of transverse colon that does not allow passage were determined. Multiple biopsies were obtained from both of the masses. The pathology reported the distal located polypoid lesion to be low differanciated mucinous adenocarcinoma and proximally located invased lesion to be an adenocarcinoma developed on the basis of a tubulovilleous adenoma.

**Figure 3:**
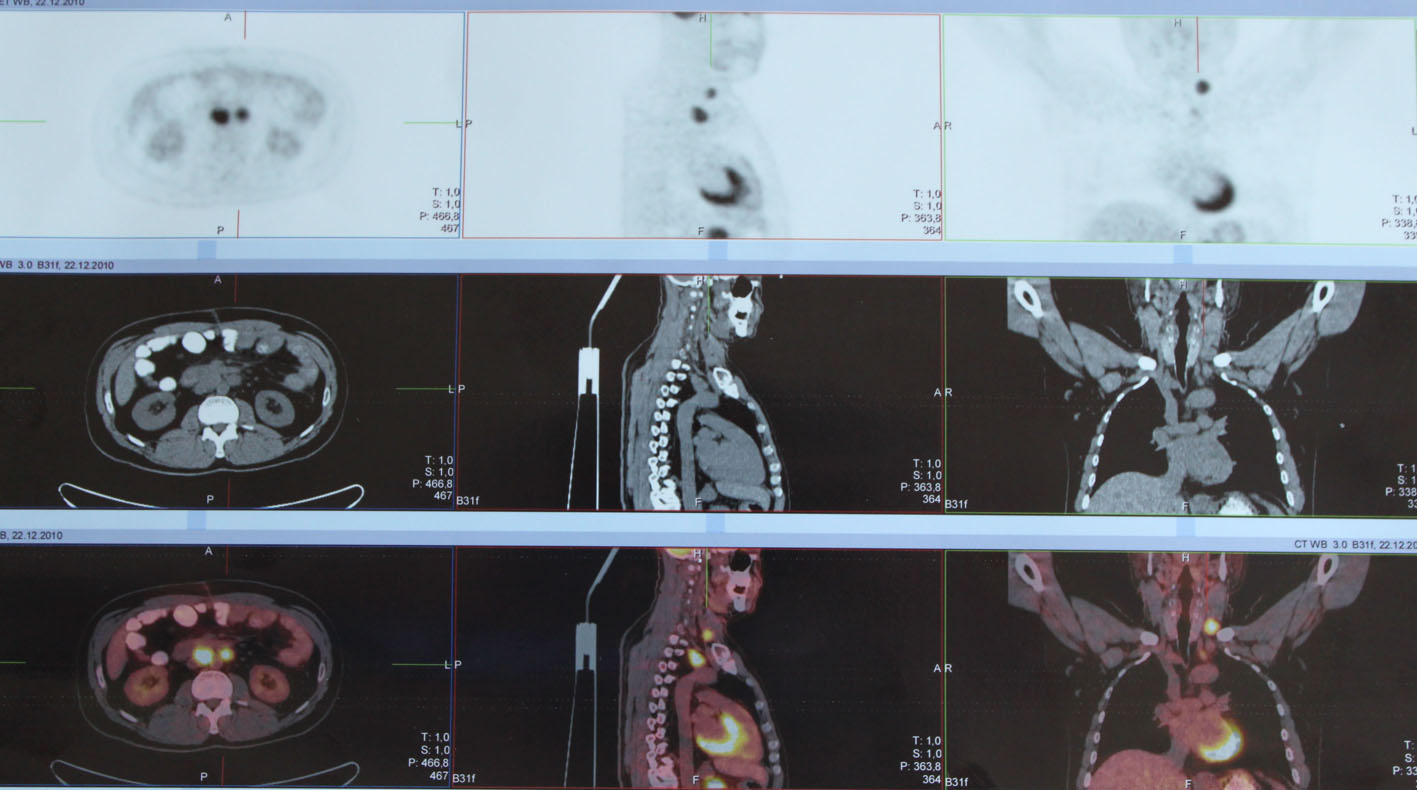
Multiple metastatic lymph node invasion in left inferior jugular region, anterior mediastinum, gastric contiguity and abdominopelvis in PET-Scan.

An operation was decided to be made. During operation, an invased tumoral mass of 12 × 15 cm on abdominal wall in the middle of transverse colon that has exceeded serosa was seen. Lymph nodes some of which over 1 cm of width were present in omentum and intestinal meso. Other intraabdominal organs looked normal macroscopically. Total proctocolectomy, ilioanal anastomosis and omentectomy were performed. In pathological report, it was declared that invasive tumor exceeded serosa and metastasis were present in seven of the lymph nodes. K-ras gene mutation was determined in genetic analysis. In PET-Scan performed before adjutant chemotherapy; multiple metastatic lymph nodes in sol inferior jugular region, anterior mediastinum and abdominopelvis were established (Fig. 3). The phase of the tumor was detected to be T_4_N_2_M_1_ according to TNM classification.

## Discussion

HNPCC are critically important for their familial transition, early onset, poor differentiation hystologically and higher frequency of syncrone and metacron relatively [2]. Although having an incidence of 2-7% in all CRC cases, up to 80% of developing cancer makes HNPCC an important circumstance in terms of patient follow up for early diagnosis [2, 4].

Male gender and age of 41 in this reported case is showing comformity with classical knowledge on HNPCC syndrome [4, 5]. Considering the less frequent left colon location for HNPCC, the case is outstanding [6]. The disease may be accompanied by extracolonic malignencies (ovaries, ureter, pelvis renalis, endometrium, stomach, intestines) [5, 7]. In our patient, there were no extracolonic organ metastasis whereas multiple metastatic lymph node were present in cervical, thoracal and abdominopelvic regions. The reason for diagnosis of the disease in later phases may be due to diagnosis of the sisters and brothers at the same period of time and delayed diagnosis of the familial transient disease.

Although syncrone colon tumors are seen in 2-4% sporadic of cases, the incidence is a little bit higher in HNPCC cases [12, 16]. The incidence of different hystopathological type in syncrone tumors is 3% and of these lesions 33-55% are villeous adenomas [12, 17]. In our case, an adenocarcinoma that developed on the basis of a tubulovilleous adenoma in transverse colon and a low differentiated mucinous adenocarcinoma in rectosigmoidal region were determined.

Tumors on HNPCC pathway show mutations on APC, p53 and K-ras genes [14]. Mutations on K-ras gene are much more rare than ones seen in CIN [15]. In this reported case, K-ras gene mutation was detected.

Early onset, lifetime risk of having cancer, high frequency of extracolonic metastasis and low differentiated hystology of CRC’s makes close follow up for HNPCC an important factor. Showing familial transition in these group of patients and follow up programs for family members under risk may introduce the chance of early diagnosis and treatment.
